# Management of Invasive Cervical Root Resorption in a Mandibular Canine Using Biodentine as a Restorative Material: A Case Report 

**DOI:** 10.22037/iej.v12i3.16668

**Published:** 2017

**Authors:** Leila Eftekhar, Hengameh Ashraf, Sanaa Jabbari

**Affiliations:** a * Department of Pediatric Dentistry, Dental School, Shahid Beheshti University of Medical Sciences, Tehran, Iran; *; b *Department of Endodontics, Dental School, Shahid Beheshti University of Medical sciences, Tehran, Iran*

**Keywords:** Biodentine, External Root Resorption, Invasive Cervical Resorption, Mandibular Canine

## Abstract

Invasive cervical root resorption (ICR) is the reversible/irreversible loss of tooth structure in the connective tissue attachment zone with unclear etiology. In the present case a history of periodontal surgery was presumed to be the predisposing factor. Early diagnosis and proper treatment may lead to long-term retention of the tooth. The tooth is usually asymptomatic and diagnosis is commonly made as a result of a routine radiographic finding. Cone-beam computed tomography (CBCT) is extremely useful in diagnosis and treatment planning of ICR. The treatment should aim toward complete removal of the resorptive defect and reconstruction by placement of a suitable filling material. The present report demonstrates the management of a mandibular canine with invasive cervical root resorption using Biodentine for the defect.

## Introduction

External root resorption is a process that leads to reversible/irreversible loss of cementum, dentin and bone [1] as a result of odontoclastic action [2]. It occurs in both vital and pulpless teeth. External resorption may be physiological or pathological [1]. Cervical external resorption *aka* invasive cervical resorption [3] or peripheral inflammatory root resorption (PIRR) [3], is a clinical term used to describe a relatively uncommon, insidious and often aggressive form of external tooth resorption [2] on the surface of root below the epithelial attachment and the coronal aspect of the supporting alveolar process, namely the zone of the connective tissue attachment [4]. Resorption of coronal dentin and enamel often creates a clinically obvious pinkish color in the tooth crown as highly vascular resorptive tissue becomes visible through thin residual enamel [5].

The etiology of invasive cervical resorption is poorly understood, several potential predisposing factors have been identified, in one study, intra-coronal bleaching has been suggested as the most widely documented factor [5] whereas in another study orthodontic treatment was the most common sole factor identified [6]. Other various predisposing factors basically include either physical (orthodontic treatment, segmental orthognathic surgery, transplanted teeth, trauma, bruxism, guided tissue regeneration) or chemical trauma (intracoronal bleaching, bone grafting, tetracycline conditioning of root) [2, 7] and periodontal treatment (including deep root scaling and planning [6]) have also been cited [5].

The condition is usually painless and diagnosis is usually the result of a routine radiologic examination [5]. Heithersay G.S. [3] has proposed a clinical classification of invasive cervical resorption depending on the amount of destruction: Class 1; a small invasive resorptive lesion near the cervical area with shallow penetration into dentin, class 2; a well-defined invasive resorptive lesion that has penetrated close to the coronal pulp chamber but shows little or no extension into the radicular dentin, class 3; a deeper invasion of dentin deeper invasion of dentin by resorbing tissue, not only involving the coronal dentin but also extending into the coronal third of the root and class 4; a large, invasive resorptive process that has extended beyond the coronal third of the root [8].

**Figure 1:**
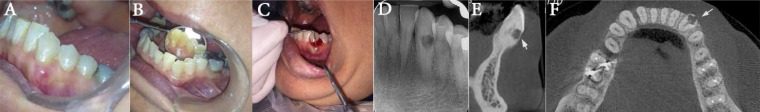
*A* and *B*) Preoperative intraoral photograph; *C)* Preoperative periodontal examination, probing depth of 6 mm and bleeding on probing; *D)* Preoperative intraoral periapical radiograph; *E* and *F)* Preoperative CBCT view of mandibular left canine on sagittal and axial sections, respectively

**Figure 2:**
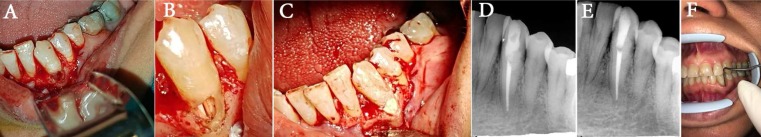
)* Cervical resorption defect seen after flap reflection; *B)* Prepared cavity and the tapered gutta-percha within root canal; *C)* Sealing the defect with Biodentine; *D)* Postoperative intraoral periapical radiograph; *E)* Postoperative radiograph after 2-year follow--up; *F)* Postoperative intraoral photograph after 2-year follow-up*

Radiographic features of lesions range from well-delineated to irregularly bordered mottled radiolucencies, which can be confused with dental caries. A characteristic radiopaque line generally separates the image of the lesion from that of the root canal, because the pulp remains protected by a thin layer of predentin until late stages of the process [8]. Cone-beam computed tomography (CBCT) is extremely useful in the diagnoses and treatment planning of invasive cervical resorption [9, 10].

The basic treatment modalities are complete removal of resorptive tissue, and then dentinal walls are conditioned by 90% aqueous solution of trichloracetic acid for coagulation necrosis of the resorptive tissue without damaging the periodontal tissue. Any undermined dentin or enamel at the peripheries of the cavity are removed with a bur in a high-speed handpiece, and the cavity is restored by appropriate restorative material like glass-ionomer cement, composite resin [2, 10] or calcium silicate-based cements [3] such as mineral trioxide aggregate (MTA) [11, 12], Biodentine (Septodont, Saint-Maur-des-Fosses, France) [13], and calcium-enriched mixture (CEM) cement [14]. Once the cavity has been restored, the mucoperiosteal flap is replaced and secured in position [10]. 

Biodentine may prove to be a particularly suitable material for restoring these defects because it may combine acceptable aesthetics with the ability to support PDL attachment [10, 15]. Therefore, the present case report describes surgical management of mandibular canine with external cervical resorption using Biodentine.

## Case Report

A 51-year-old female with a chief complaint of pain in the lower-anterior portion of her gums during past two weeks presented to Endodontic department, Dental School, Shahid Beheshti University of Medical Sciences. Medical history of the patient was noncontributory. The patient mentioned a history of recurrent localized swelling during past three months in buccal mucosa of mandibular left canine. Past dental history revealed that she had undergone periodontal surgery in anterior left segment of mandible two years earlier.

On clinical examinations, the buccal gingiva of tooth was sensitive to palpation .The sensitivity tests including electric pulp test and thermal tests were negative while the adjacent tooth responded normally. On periodontal examination probing depth was approximately 6 mm on buccal sulcus of tooth and bleeding on probing was obvious. Tooth was not mobile and no caries or previous filling was present (Figures 1A to C).

On radiographic examination, radiolucency in cervical area and coronal third of the root was present and normal periradicular and periodontal ligament status was seen. CBCT images confirmed the presence of external cervical root resorption on buccal surface of the root and perforation of root canal wall (Figures 1C to E).

A diagnosis of pulp necrosis with external cervical root resorption (Heithersay’s type III) was made, therefore root canal therapy and surgery for sealing the resorptive defect was planned.

Written informed consent was obtained from the patient. Under local anesthesia, buccal and lingual infiltration of 2% lidocaine containing 1:80000 epinephrine (Darupakhsh, Tehran, Iran) was administered and rubber dam was placed. Access cavity was prepared on lingual surface. After working length determination with #15 K-file (Mani, Tochigi, Japan), cleaning and shaping of root canal was performed using ProTaper rotary system (Dentsply, Tulsa Dental, Tulsa, OK, USA). S1, S2, F1 and F2 rotary files were employed up to the working length. At first visit, irrigation was performed with normal saline solely, due to the presence of the root canal perforation. During the cleaning and shaping process, profuse bleeding started, possibly from perforated area. Since proper cleaning and debridement of root canal system before complete removal of resorptive tissue and sealing the perforation area was not possible, the tooth was not obturated in first visit. Mild bleeding was still present at the end of the cleaning and shaping process. Root canal was partially dried with paper points and a tapered gutta-percha (#30/0.04) was placed in canal to maintain its patency during the restoration process in forthcoming surgery. The access cavity was then sealed with Coltosol (Speedex; Coltene, Altstatten, Switzerland).

At second visit after administration of buccal and lingual infiltration, an envelope full mucoperiosteal flap was reflected (Figure 2A). Granulomatous tissue was excavated from the resorptive area using hand excavator. The cavity was treated with 90% aqueous solution of trichloracetic acid. After removing any undermined dentin or enamel with a bur in a high speed handpiece (Figure 2B) and drying the prepared cavity, the resorptive area was filled with Biodentine (Septodont, Saint-Maur-des-Fosses, France) and contoured properly (Figure 2C). A periapical radiography was taken to confirm proper restoration of the resorptive area. Finally, the flap was repositioned to its original position and sutured with 3-0 black silk suture material. Chlorhexidine oral rinse 0.12% was administered (twice daily for 7 days). Sutures were removed 4 days later. The root canal cleaning and shaping was completed 10 days after surgery by irrigating with 2.5% hypochlorite sodium and the canal was obturated using gutta-percha and AH-26 sealer (Dentsply, Tulsa Dental, Tulsa, OK, USA) (Figure 2D). The access cavity was restored with resin composite one week later.

Clinical and radiographic examinations were performed for treated teeth after 6, 12 and 24 months. Patient was completely asymptomatic and probing depth was within normal limits at each follow-up time point indicating that repair of resorption defect was successfully performed with Biodentine (Figures 2E and F).

## Discussion

The presented case describes an invasive cervical root resorption; the tooth required root canal treatment due to perforation of root canal wall and pulp necrosis, followed by sealing the resorptive area with Biodentine. The etiological factor for the resorption in this case is periodontal treatment which resulted into resorptive defects. Root resorption in permanent dentition has pathological basis and is classified into internal and external resorption. Cervical external root resorption also called as invasive cervical resorption (ICR) is a localized resorptive process that commences on the surface of the root below the epithelial attachment and the coronal aspect of the supporting alveolar process and is symptomless until the destruction reaches the pulp [16]. ICR has various etiologic factors such as intra-coronal bleaching, orthodontic treatment, bruxism, periodontal treatment, bone grafting [2, 5]. ICR is classified into four classes according to the extent and severity of the lesion [8]. ICR is usually an incidental radiographic finding and is classically an asymmetrical radiolucency with ragged or irregular margins in the cervical region of the tooth [16]. CBCT is extremely useful in the diagnoses and treatment planning of ICR [10].

There are different modalities for treatment of external cervical root resorption. Treatment alternatives are case-dependent and depends on the etiology of resorption. The basic aim of treating is the complete removal of resorptive tissue and the restoration of the defected area. The basic treatment modalities are flap reflection, complete removal of resorptive tissue, restoration of the cavity by appropriate restorative material like glass-ionomer cement, composite resin [3], calcium silicate-based cements [3] such as mineral trioxide aggregate (MTA) [17], Biodentine (Septodont, Saint-Maur-des-Fosses, France) [16], and calcium-enriched mixture (CEM) cement [17]. Once the cavity is restored, the mucoperiosteal flap is replaced and secured in position [10].

Biodentine is a new tricalcium silicate (Ca_3_SiO_5_) based inorganic restorative cement. Hydraulic calcium silicate cements are bioactive materials showing a dynamic interaction with dentin and pulp tissue interface and they can stimulate pulpal cell recruitment and differentiation. They also, up-regulate transformation factors and promote dentinogenesis [18]. Its property to release calcium ion and enhancing the alkaline environment makes Biodentine more conducive for osteoblastic activity [18, 19].

Biodentine has a wide range of applications including endodontic repair (root perforations, apexification, resorptive lesions and retrograde filling material in endodontic surgery) and pulp capping and can be used as a dentine replacement material in restorative dentistry [15]. The material is claimed to possess better physical and biological properties compared to other tricalcium silicate cements such as mineral trioxide aggregate (MTA) and Bioaggregate [18]. Teeth treated with white MTA exhibited discoloration, whereas those treated with Biodentine maintained color stability [20]. Biodentine has better material handling properties compared to MTA, which is more time consuming and technically difficult [18]. Biodentine has setting time of less than 12 min and high mechanical properties with excellent sealing ability [18, 19]. 

In the case presented the diagnosis was Heithersay’s class III ICR. The treatment plan was combination of surgical approach and non-surgical root canal treatment. The selected material was Biodentine because of better consistency after mixing, better handling properties, faster setting and also less tooth discoloration, compared to MTA. It is bioactive, biocompatible, and non-resorbable and having sufficient amount of push out bond strength with dentinal walls which makes it an excellent material for perforation repair. Also it produces tighter seal in the area of the defect and prevents microleakage [21]. After the treatment, the patient attended regular visit for check-up. Proper healing of the defect was observed during a 2-year of follow-up. The patient was asymptomatic and the radiographic examinations showed no signs of periradicular pathology during follow-up period.

## Conclusion

Early diagnosis, correct case selection, an appropriate restorative/regenerative material and proper treatment are essential for long-term retention of the tooth with invasive cervical root resorption. Although this case report presents a favorable outcome, further studies are encouraged to support the use of Biodentine to fill external ICR defect.
